# Development of a mixed microbial culture for robust high-throughput ex-situ thermophilic biomethanation

**DOI:** 10.3389/fmicb.2026.1840981

**Published:** 2026-05-28

**Authors:** Rajkumar Gangappa, Savvas Savvas, Sky Redhead, Tim Patterson, Sandra Esteves

**Affiliations:** 1 Wales Centre of Excellence for Anaerobic Digestion, Sustainable Environment Research Centre, Faculty of Computing, Engineering and Science, University of South Wales, Pontypridd, United Kingdom; 2 Faculty of Computing, Engineering and Science, University of South Wales, Pontypridd, United Kingdom

**Keywords:** ex-situ biomethanation, mixed microbial culture, power to methane, sustainable fuel, thermophiles

## Abstract

Ex-situ biomethanation using mixed microbial cultures is a promising approach for carbon capture and utilisation, converting CO₂ and renewable H₂ into grid-compatible methane. However, achieving high H₂/CO₂ throughputs at ambient pressure remains constrained by microbial competition and process instability. This study presents a novel operational strategy integrating autoclave pretreatment, targeted microbial conditioning, and intermittent washouts to achieve stable, high throughput ex-situ biomethanation using autoclaved, microbe rich sludge.

Autoclave pretreatment of seed sludge reduced total bacteria by ~92.5%, while key methanogens, *Methanobacteria*, *Methanosarcina*, and *Methanosaeta,* were declined by 91.5, 85, and 77%, respectively, but not fully eliminated. This non-specific biomass reduction enabled controlled management of a resilient mixed consortium, promoting rapid adaptation and enhanced methanogenic performance under high H_2_/CO_2_ throughputs.

At 37 °C and H₂/CO₂ throughput of 450 L/L/d, methane content remained below 60%. In contrast, conditioning the microbial community at 50 °C under a stoichiometric H₂: CO₂ ratio of 4:1 significantly enhanced performance, increasing methane content to 82%. Further increasing the throughput to 500 L/L/d raised methane content to 86%, with potential to exceed 90%. This represents the highest reported throughput and methane quality in continuously stirred tank reactors operating under atmospheric pressure and 50 °C. The optimised system developed a stable consortium dominated by *Euryarchaeota* (78%), alongside *Firmicutes* (16%) and *Coprothermobacterota* (4%). Intermittent washouts effectively regulated metabolic intermediates, stabilising volatile fatty acids at ~3.5 g/L, primarily acetate (73%).

Overall, this study demonstrates a robust strategy for high-throughput ex-situ biomethanation, advancing CO₂ valorisation and supporting energy storage and circular carbon management.

## Graphical abstract

**Figure:**
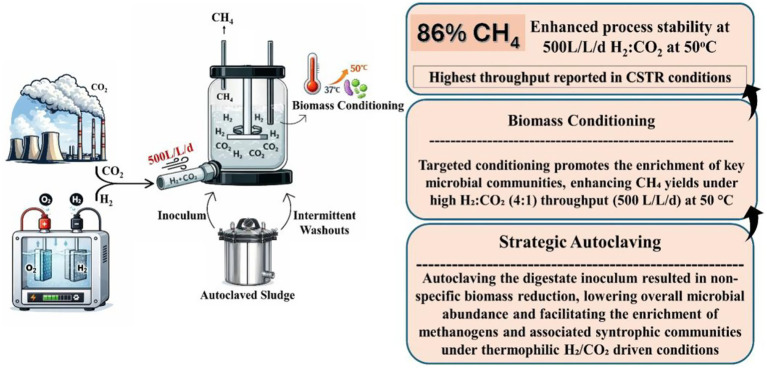

## 1 Introduction

Ex-situ biomethanation is an emerging technology used for converting carbon dioxide (CO_2_) and renewable hydrogen (H_2_) into biomethane (CH_4_) as a fuel, sometimes referred to synthetic methane or e-methane. This technology utilises methanogenic archaea to convert CO_2_ into CH_4_ through hydrogenotrophic methanogenesis (Patterson et al., 2017), which can be performed with either pure cultures or mixed microbial cultures (MMCs).

MMCs, such as those found in anaerobic digestate are considered more resilient and economical compared to pure cultures for converting H_2_ and CO_2_ into CH_4_ (Xu et al., 2020; Savvas et al., 2018). However, they may induce competition for shared substrates like H_2_ and CO_2_, potentially leading to the accumulation of unwanted by-products and instability in the ex-situ biomethanation process (Savvas et al., 2018; Tsapekos et al., 2022; Logroño et al., 2020; Dar et al., 2008). Some reported strategies to mitigate this competition have been considered largely for anaerobic digestion of organic wastes rather than H_2_/CO_2_ conversions. These include the use of additives to enhance hydrolysis, nutrient supplements, bio-augmentation and co-digestion (Das et al., 2023; Li et al., 2020; Ye et al., 2018; Boonyakitsombut et al., 2002). For H_2_/CO_2_ conversions, intermittent hydrogen starvation and flexible H_2_/CO_2_ feeding strategies have been explored (Thapa et al., 2022; Logroño et al., 2021). However, these methods require adjustment based on the process and type of the starting inoculum, as no single approach fits all conditions.

In conventional anaerobic digestion of organic wastes, the microbial community is diverse, including both fermentative bacteria and methanogens, and the goal is to enhance the breakdown of a wide range of organic compounds. However, in ex-situ biomethanation, even when using anaerobic digestate derived inoculum, the process selectively promotes hydrogenotrophic methanogens that specialise in converting H₂ and CO₂ into CH₄. Therefore, strategies designed to enhance anaerobic digestion of organic wastes may not be effective for ex-situ biomethanation unless they specifically promote key methanogens. Hence, optimising ex-situ biomethanation with MMCs requires management of bacterial communities, especially those that compete with methanogens for H_2_ and CO_2_. This regulation can reduce resource competition and streamline syntrophic interactions.

### 1.1 Competition for shared resources

In ex-situ biomethanation with continuous H₂ and CO₂ supply, MMC sub-communities including sulphur reducing bacteria (SRB), methanogens, and homoacetogens, compete for these gases, leading to various end products (Dar et al., 2008). SRBs dominate with excess sulphate and limited H₂, while methanogens prevail when sulphate is minimal. However, under high H₂ partial pressure, homoacetogens can outcompete methanogens, consuming up to 40% of the available H₂ and rapidly producing volatile fatty acids (VFAs), which are detrimental to CH₄ production (Tsapekos et al., 2022; Saady, 2013; Stams et al., 2005). Moreover, it has been stated that VFA accumulation can lower pH below 6.0, inhibiting methanogens and risking process failure (Logroño et al., 2020). To address this microbial imbalance, autoclave pretreatment of seed digestate may be an effective strategy. This pretreatment could non-specifically suppress or eliminate non-spore forming bacteria, shifting the balance towards methanogens (Tampio et al., 2015), and improving CH₄ yield.

### 1.2 Microbial syntrophic interactions

Ex-situ biomethanation with MMCs relies on complex interactions between bacterial communities and methanogens, known as syntrophic relationships. These interactions involve intricate biochemical exchanges that enable the breakdown of organic compounds and the generation of CH₄ (Kumar et al., 2021). However, the complexity of these interactions can challenge the stability and predictability of the biomethanation process, particularly under high H_2_/CO_2_ throughput conditions.

Autoclave pretreatment of digestate inoculum could potentially offer solution by simplifying microbial interactions. The reduction in bacterial diversity may streamline the syntrophic network between spore forming bacteria and methanogens, leading to a more efficient ex-situ biomethane production pathway. Also, the reduced microbial complexity could improve the stability of the biomethanation process, making it more predictable and reliable.

### 1.3 High throughput H₂/CO₂ conversion via ex-situ biomethanation

Despite advancements in ex-situ biomethanation using MMCs, a significant research gap remains in exploring high H₂/CO₂ throughputs under ambient pressure conditions. A major challenge lies in maintaining microbial balance within complex inocula like anaerobic digestate, where competition between hydrogenotrophic methanogens and bacteria can cause VFA accumulation, inhibiting methanogenesis.

Reported H_2_/CO_2_ throughputs for ex-situ biomethanation using MMCs typically range between 70 and 360 L/L/d (Savvas et al., 2024; Sposob et al., 2021; Strübing et al., 2019; Dupnock and Deshusses, 2017; Savvas et al., 2018). This study aimed to substantially exceed those values by targeting a throughput of 500 L/L/d. Moreover, the study investigates whether methanogenic activity and process stability can be sustained at such high throughput under thermophilic conditions. Mesophilic anaerobic processes are generally understood to have lower energy demands and greater process stability when compared to thermophilic operation. However, thermophilic anaerobic processes can provide benefits such as accelerated biochemical reactions, faster microbial growth rates, enhanced interspecies H_2_ transfer, and increased destruction of pathogenic bacteria, thereby increasing CH_4_ production potential even with shorter hydraulic retention times (Gavala et al., 2003).

This study presents a novel integrated strategy combining autoclave pretreatment, microbial conditioning, and intermittent washouts to optimise high throughput ex-situ biomethanation. Autoclave pretreatment was employed to non-specifically supress competing bacteria and enrich key methanogens, while microbial conditioning was designed to facilitate adaptation of thermophilic consortia to support high H₂/CO_2_ throughputs. Intermittent culture washouts were introduced as a control strategy to limit VFA buildup and maintain community stability, thereby, enhancing overall system stability. The work further examines the influence of temperature by comparing mesophilic and thermophilic H₂/CO₂ conversion under high-throughput conditions, providing insights into process performance and operational robustness. Overall, the study aims to inform strategies for achieving high rate biomethanation, targeting H₂/CO₂ feed rates of up to 500 L/L/d, and contributing to the development of decentralised renewable energy storage systems.

## 2 Materials and methods

### 2.1 Reactor configuration and inoculum

Ex-situ biomethanation was carried out in a custom-designed Continuous Stirred Tank Reactor (CSTR) with a total volume of 1.73 L and a working volume of 0.4 L. The reactor was optimized for efficient gas–liquid mass transfer (kLa) through high-speed mixing using a 60 mm Rushton-type impeller with six flat blades operated at constant stirring speed of 2,500 rpm throughout the experiment, ensuring optimal gas distribution and minimal bubble size. The CSTR measuring 220 mm in height and 100 mm in internal diameter, was equipped with an external heating pad which was controlled using an Elitech STC-1000 controller.

Anaerobic digested sewage sludge, sourced from a local wastewater treatment multi-stage mesophilic digestion plant, was used as the inoculum. Prior to use in the experiments the digestate was filtered using 300 μm sieve and subsequently was autoclaved at 121 °C and pressure of 15 psi for 1 h. The final inoculum contained about 2% total solids (TS) and 1% volatile solids (VS). The experiments were conducted in a single CSTR as a proof -of-concept system to evaluate microbial enrichment and process performance under high H_2_/CO_2_ throughput conditions. Also, as this study focused on ex-situ hydrogenotrophic biomethanation, conventional wastewater or anaerobic digestion parameters such as COD, TOC, and TKN were not monitored, as CH_4_ production was primarily driven by gaseous substrates (H₂/CO₂).

H₂ and CO₂ gases were supplied from pressurised gas cylinders (>99.9% purity, Air Liquide, United Kingdom). They were pre-mixed at various stoichiometric ratios using Alicat M-Gas mass flow meters (UK). These meters have a 10,000:1 turndown ratio, with full-scale flow rates ranging from 0.5 SCCM (Standard Cubic Centimeters per Minute) to 5,000 SLPM (Standard Litres per Minute) and maximum operating pressure of 145 PSIG (Pounds per Square Inch Gauge). Additionally, they offer high repeatability, with calibrations up to ±0.6% of the reading accuracy.

### 2.2 Experimental regime

The experiment was divided into four distinct phases.

#### 2.2.1 Phase 1 (days 0–40): Operation at mesophilic conditions

Phase 1 of the experiment was undertaken at mesophilic temperature (37 °C). The reactor was loaded with 0.4 L of autoclaved digestated sludge, an initial H₂/CO₂ input of 45 L/L/d at a stoichiometric ratio of 4:1 was supplied, and the reactor contents were stirred at 2500 rpm. Operational adjustments included intermittent washouts, during which 50% of the culture was replaced with fresh autoclaved sludge. These washouts were primarily determined by a drop in pH (< 6.5) or a low CH_4_ percentage (< 60%) in the output gas. After each washout, the H₂/CO₂ throughput rates were progressively increased while keeping the temperature and stirring rate constant. By day 25, the H₂/CO_2_ throughput reached 450 L/L/d, a rate that was sustained until the end of this phase on day 40.

#### 2.2.2 Phase 2 (days 41–49): Thermophilic conditioning of biomass

Temperature conditioning of the biomass was initiated to evaluate the impact of temperature adjustments on CH₄ synthesis and process stability. Starting on day 41, the temperature was gradually increased by 2–3 °C every 24 h following a 50% culture washout. This systematic temperature increase continued until the temperature reached 55 °C by the end of the phase (day 49). During this period, the H_2_/CO_2_ throughput and stirring speed were consistently maintained at 450 L/L/d and 2,500 rpm, respectively.

#### 2.2.3 Phase 3 (days 50–63): Fine tuning H_2_/CO_2_ ratio for stable biomethanation

After the biomass conditioning to higher temperatures in phase 2, the H₂/CO₂ (4:1) throughput was increased to 500 L/L/d at 50 °C. However, this adjustment initially disrupted CH₄ production, necessitating H_2_ input optimisation to restore process stability, while the total gas inflow was maintained at 500 L/L/d. On day 56, the H_2_: CO_2_ ratio was reduced to 3:1 following a 50% washout. Subsequently, the H₂ fraction in the gas mixture was increased by 0.1 or 0.2 units every 24 h after each 50% washout. By day 63, the original H₂: CO₂ ratio of 4:1 was successfully re-established.

#### 2.2.4 Phase 4 (days 64–69): Testing conditioned biomass for high throughput ex-situ thermophilic biomethanation

The final phase focused on assessing the performance of the optimised biomass under elevated temperature and enhanced H₂/CO₂ throughput in steady conditions. The temperature was maintained at 50 °C, with a H_2_: CO2 (4:1) throughput of 500 L/L/d. Modifications to the washout process included reducing the washout percentage from 50 to 25% while continuing daily washouts. This adjustment aimed to evaluate the recovery speed of CH₄ production without disrupting the biomethanation process.

### 2.3 Gas measurements, VFAs analysis, and pH recording

Real-time measurements of CH₄ and CO₂ levels in the output gas was conducted using infrared sensors (0–100% v/v detection range) from Edinburgh Sensors Ltd. A Restek ProFlow 6,000 electronic flowmeter (UK) was used to quantify volumetric gas output, offering a measurement range of 0.5–500 mL/min and an accuracy of ±0.2 mL/min. Sample pH was determined with a Hanna Instruments pH electrode (HI-1286) and transmitter (BL-931700).

The concentration and composition of VFAs were analysed using a method adapted from Cruwys et al. (2002). A Perkin Elmer Autosystem XL gas chromatograph equipped with a flame ionization detector and a Supelco column (30 m × 0.32 mm) was employed for this analysis, using N₂ as the carrier gas.

### 2.4 16S rRNA sequence analysis via nucleic acid sequencing and quantitative polymerase chain reaction

#### 2.4.1 DNA extraction and quantitative polymerase chain reaction

DNA was extracted using the Exgene Soil DNA kit (GeneALL Biotechnology Ltd.) and quantified with a Qubit-4 fluorometer (Qubit® dsDNA-HS Assay Kit, Life Technologies, United States). Total bacterial gene copies were determined using bacterial rDNA standards (Suzuki et al., 2000), while hydrogenotrophic and acetoclastic methanogens were quantified according to (Yu et al., 2005). Positive controls included DNA from pure cultures obtained from the Leibniz Institute DSMZ, with *Halorubrum saccharovorum* (DSM 1137) used as a bacterial control. Methanogenic populations were assessed using species *Methanosaeta concilii* (DSM 6752), *Methanosarcina barkeri* (DSM 800), and *Methanobacterium bryantii* (DSM 863). The qPCR analysis was conducted using the Roche LightCycler Nano, employing TaqMan primers and probes (Thermo Scientific, United Kingdom). The calibration was performed using standards and the qPCR method, as described by Savvas et al. (2017).

#### 2.4.2 Bacterial and archaeal community analysis

Microbial community analysis was performed on samples collected from the reactor, with DNA extracted as described in the section 2.4.1 and quality was determined using the Qubit-4 fluorometer. High-throughput sequencing was performed on the HiSeq Illumina platform with bacterial (515F/806R) and archaeal (Arch519F/Arch915R) 16S rRNA gene primers (Novogene, UK). Sequence data was processed with QIIME-2 to pair sequences and remove chimeric sequences using UCHIME algorithm. Operational taxonomic units (OTUs) were clustered at 97% similarity with UCLUST algorithm, and taxonomic assignment was done using Mothur software.

### 2.5 Bacterial community analysis of autoclaved and original sludge

#### 2.5.1 Serial dilution and plating

A tenfold serial dilution of autoclaved and original sludge samples was prepared using phosphate buffered saline (PBS, 1X, pH 7.4). Diluted samples were plated onto nutrient agar (Oxoid) and incubated at 30 °C for 48 h under microaerophilic conditions. Colony-forming units (CFUs) were assessed based on their morphology (Jackman, 2011).

#### 2.5.2 Isolation of distinct colonies

Distinct colonies were isolated by streaking onto nutrient agar and incubating at 30 °C for 24 h or until colonies were visible, under microaerophilic conditions.

#### 2.5.3 16S rRNA gene amplification and sequencing

Colony PCR targeting the 16S rRNA gene was performed on morphologically distinct colonies using a mix of 15 μL PCR Mastermix (Promega), 10 μL nuclease-free water (Promega), and 2.5 μL each of forward (5’AGA GTT TGA TCM TGG CTC AG’3) and reverse primers (5’GGT TAC CTT GTT ACG ACT T’3) (Lane, 1991). The PCR protocol included an initial denaturation at 94 °C for 5 min, followed by 30 cycles of denaturation at 94 °C for 1 min, annealing at 55 °C for 1 min, and extension at 72 °C for 2 min, with a final extension at 72 °C for 5 min. Amplified products were sent to Eurofins Genomics for Sanger sequencing. Results were analysed using Chromas v2.6.6 (Technelysium) and NCBI BLAST.

### 2.6 Scanning electron microscopy analysis

To prepare autoclaved anaerobic digestate samples for Scanning Electron Microscopy (SEM) imaging, density gradient centrifugation was employed. A Percoll solution (20.6 mL, density: 1.13 g/mL) was mixed with supernatant from ultracentrifuged digestate (79.4 mL) to match the osmotic pressure of the sludge biomass.

A 2 mL aliquot of autoclaved digestate was added to 10 mL of the prepared Percoll suspension, thoroughly mixed and centrifuged at 2,000 g for 10 min to separate the biomass. The separated biomass was stabilised at 4 °C for 30 min, then carefully pipetted out, washed with sterile PBS, and prepared for downstream analysis.

For SEM imaging, the washed biomass was fully dehydrated at 30 °C, gold-coated, and examined using an environmental SEM (Zeiss Sigma) under high-vacuum mode operating at voltage range 0.2–30 kV and current between 4pA and 100 nA.

## 3 Results and discussion

### 3.1 Managing microbial populations in the digestate through intense autoclave pre-treatment

**Figure 1:**
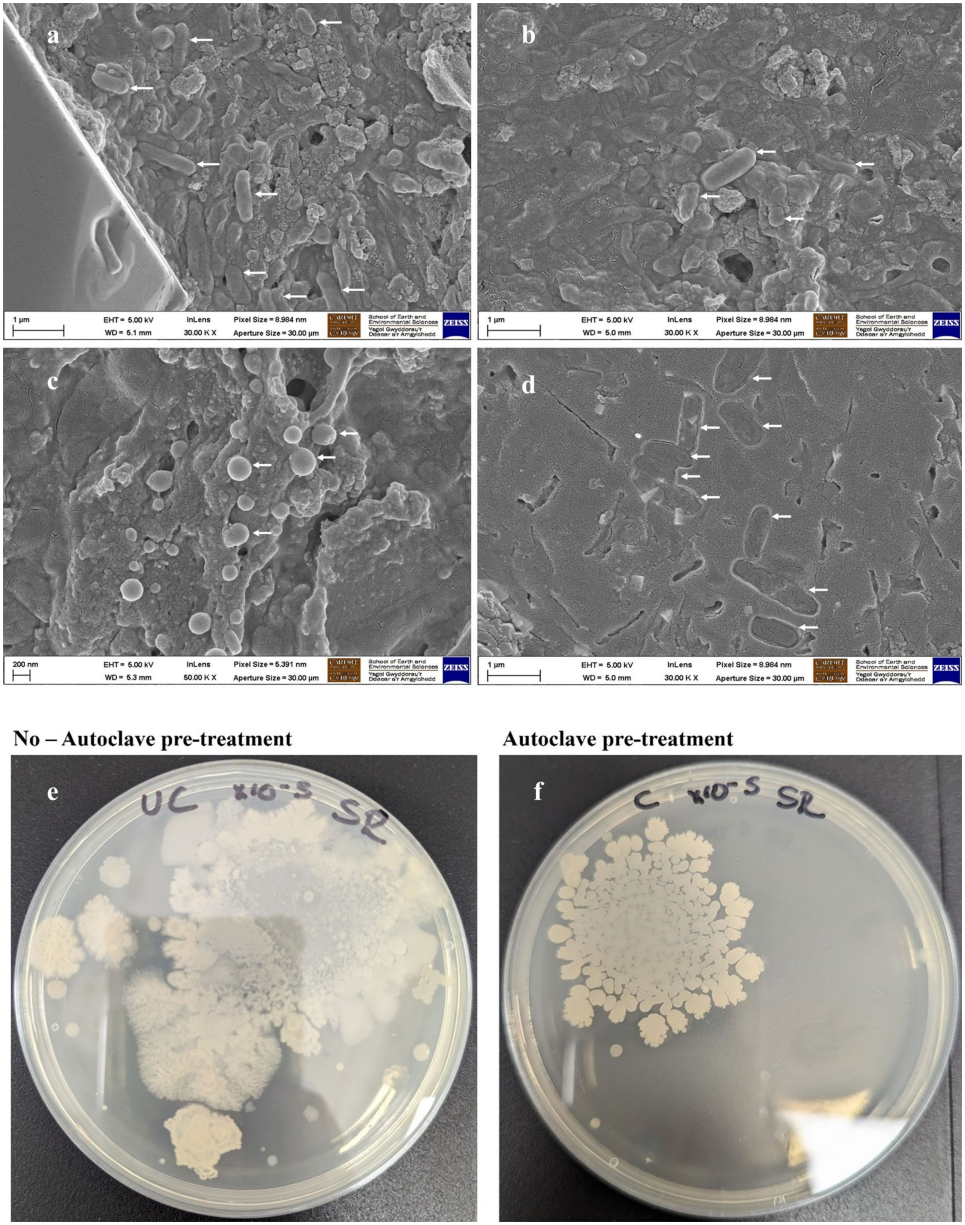
**(a,b)** SEM images of autoclaved sludge showing rod-shaped, putative bacterial cells–indicated by white arrows, measuring up to ~1 μm in length, captured at 30,000x magnification. **(c)** SEM image showing putative bacterial spores measuring up to ~200 nm, captured at 50,000x magnification. **(d)** SEM image of bacterial cells embedded within a sludge matrix, with cell sizes exceeding 1 μm, captured at 30,000x magnification. **(e)** Bacterial colonies grown on nutrient agar from untreated sludge (no autoclave), incubated at 30 °C under microaerophilic conditions. **(f)** Bacterial colonies grown on nutrient agar from autoclaved anaerobic sludge, incubated at 30 °C under microaerophilic conditions.

SEM images of autoclaved digestate (Figures 1a–d) revealed intact microorganisms, primarily bacteria (Figures 1a,b) and their spores (Figure 1c). The bacterial cells were often observed encased within the digestate matrix (Figure 1d). Thus, given the heat resistance of bacterial spores (Setlow and Christie, 2023) and the potential shielding properties of digestate, certain bacteria within it might survive prolonged heat sterilisation procedure.

To assess the effects of autoclave treatment on microbes in digestate, bacterial populations in both pre- and post- autoclave treated sludge were compared by cultivating them on nutrient agar media under microaerophilic conditions at 30 °C (Figures 1e,f). The results showed that while autoclaving significantly reduced bacterial populations, it did not achieve complete sterilisation of the digestate. This was evident from the growth of bacterial colonies from the autoclaved sludge (Figure 1f), although at substantially lower densities than in the untreated sludge (Figure 1e). These findings suggest that while autoclaving is generally effective at inactivating or eliminating most microorganisms, its efficiency may be limited in complex matrices such as anaerobic digestate.

Anaerobic digestate consists of a mixture of partially degraded organic matter, inorganic substances, and microbial biomass, with high moisture content (Roopnarain et al., 2023). Therefore, when autoclaved, the digestate’s high organic content, viscosity, and particulate matter can create microenvironments with uneven heat and pressure distribution, shielding heat-tolerant microbes from the full sterilising effects of autoclaving.

The cultures grown on solid agar medium before and after autoclaving (Figures 1e,f) were further analysed by 16S rRNA sequencing. The plate cultures, incubated under microaerophilic conditions, revealed that from the original digestate, *Bacillus amyloliquefaciens*, *Ferdinandcohnia humi*, *Bacillus* sp., unspecified *Bacillus* sp., *Oceanobacillus caeni*, and *Bacillus aerius* proliferated. However, after autoclaving, only three bacterial species survived the intense heat and pressure: *Bacillus amyloliquefaciens*, *Ornithinibacillus* sp., and *Oceanobacillus caeni.*

*Bacillus amyloliquefaciens* is recognised as one of the most heat-resistant organisms, with its spores capable of surviving dry heat up to 420 °C (Beladjal et al., 2018). *Ornithinibacillus* sp., an endospore-forming bacterium, has been reported to be isolated from pasteurised milk (Mayr et al., 2006). Similarly, *Oceanobacillus caeni* is a spore-forming bacterium that could potentially withstand autoclaving conditions (Nam et al., 2008). Notably, samples were plated both before and after autoclaving, and resulting colonies were identified by 16S rRNA sequencing to enable direct comparison of the effects of autoclave pretreatment. As sequencing was performed on cultured colonies, the results represent viable and culturable microorganisms only, indicating bacteria that survived autoclaving and excluding contributions from DNA of non-viable cells.

In contrast to plated samples, the seed sludge before and after autoclaving was also analysed directly using qPCR and 16S rRNA sequencing. These approaches reflect the total microbial DNA pool; therefore, in autoclaved samples, the detection of DNA from cells killed or inactivated during autoclaving cannot be ruled out.

**Figure 2:**
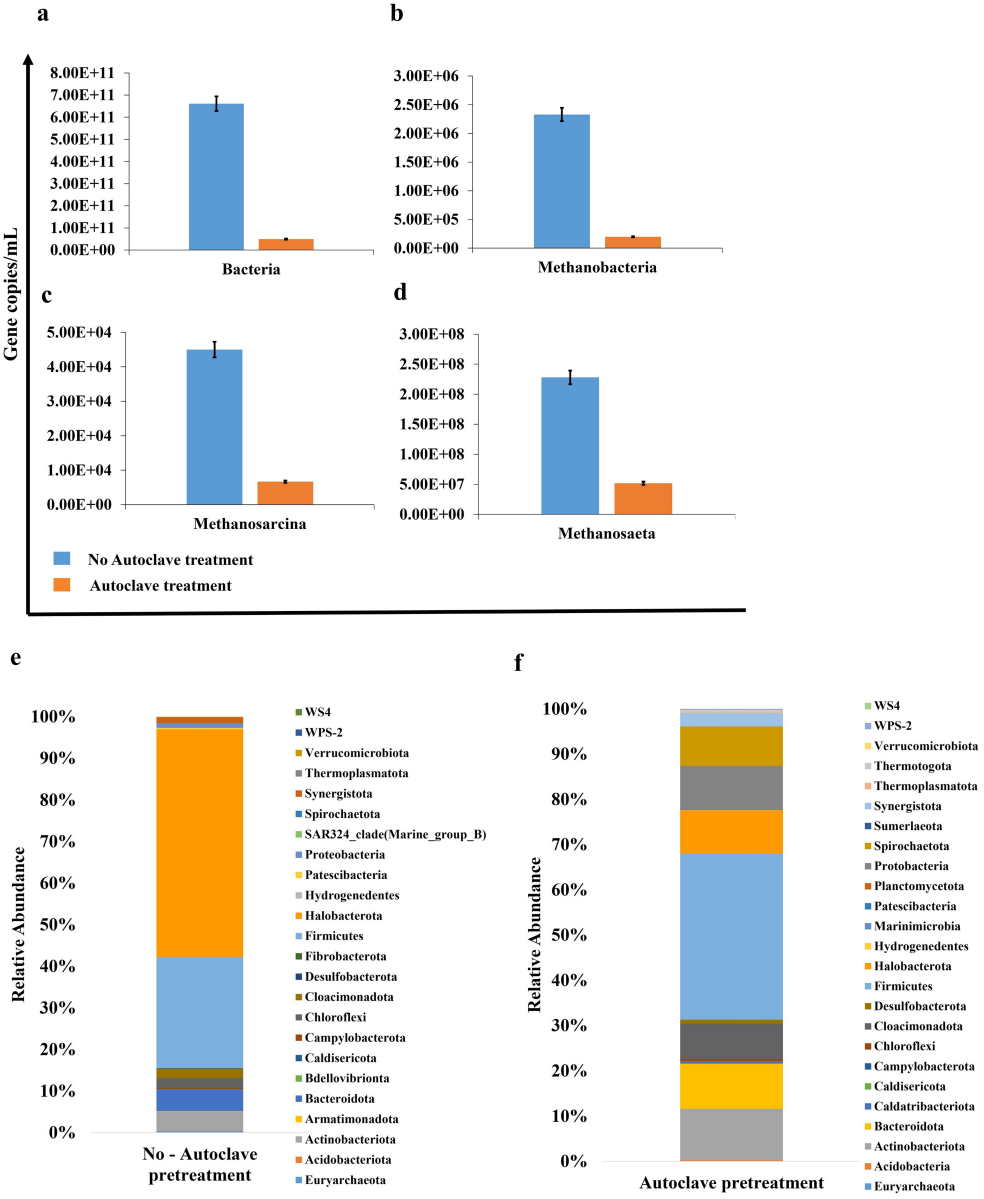
qPCR results displaying gene copy numbers per mL for bacteria **(a)**, *Methanobacteria* **(b)**, *Methanosarcina* **(c)**, and *Methanosaeta* **(d)** in untreated (blue columns) and autoclaved (orange columns) anaerobic digestate. The standard error of the mean was generally within 5% of the mean across all measurements (*n* = 3). **(e)** 16S rRNA analysis illustrating the relative abundance of microbial phyla in untreated anaerobic digestate. **(f)** 16S rRNA analysis showing the relative abundance of microbial phyla in autoclaved anaerobic digestate.

As shown in Figures 2a–d, qPCR analysis indicated ~92.5% reduction in bacterial gene copies/mL following autoclaving (Figure 2a). Methanogenic populations also declined significantly, with *Methanobacteria*, *Methanosarcina*, and *Methanosaeta* reduced by 91.5, 85, and 77%, respectively (Figures 2b–d). Despite these substantial reductions, methanogens were not fully eliminated and persisted alongside heat-resistant spore-forming bacteria.

Figures 2e,f illustrates the results of 16S rRNA gene sequencing. Initially, the microbial communities in the original sludge were predominantly characterised by the phyla *Halobacterota* and *Firmicutes*, which accounted for 54.7 and 26.7% of the total read abundance per sample, respectively. Other phyla included *Actinobacteriota*, *Bacteroidota*, *Chloroflexi*, *Cloacimonadota*, *Proteobacteria*, *Spirochaetota*, and *Synergistota*.

Following autoclave treatment, the dominant phyla were *Firmicutes*, *Actinobacteriota*, *Bacteroidota*, *Halobacterota*, *Proteobacteria*, *Spirochaetota*, *Cloacimonadota*, and *Synergistota*, comprising 36.7, 11.3, 10, 9.6, 9.5, 8.7, 7.8, and 3% of the total read abundance per sample, respectively. *Halobacterota* decreased in abundance, while relative abundance of *Firmicutes*, *Actinobacteriota*, *Bacteroidota*, and *Proteobacteria* increased. The archaeal phylum *Euryarchaeota* showed a substantial decline, decreasing from approximately 0.4% before autoclaving to 0.0062% after treatment. Overall, these results demonstrate that autoclaving significantly reshaped the microbial community structure in the seed sludge. These findings further indicate that certain microbial communities, including methanogens within anaerobic digestate can withstand the extreme conditions of an hour-long steam sterilisation. This suggests that autoclave pre-treatment can serve as a strategy to modulate methanogenic and bacterial communities within MMCs, supporting their application in ex situ biomethanation systems.

### 3.2 CH_4_ generation, microbial composition, VFAs synthesis, and pH profile

#### 3.2.1 Phase 1: Initial CH₄ production at 37 °C under varying H₂/CO₂ throughputs

**Figure 3:**
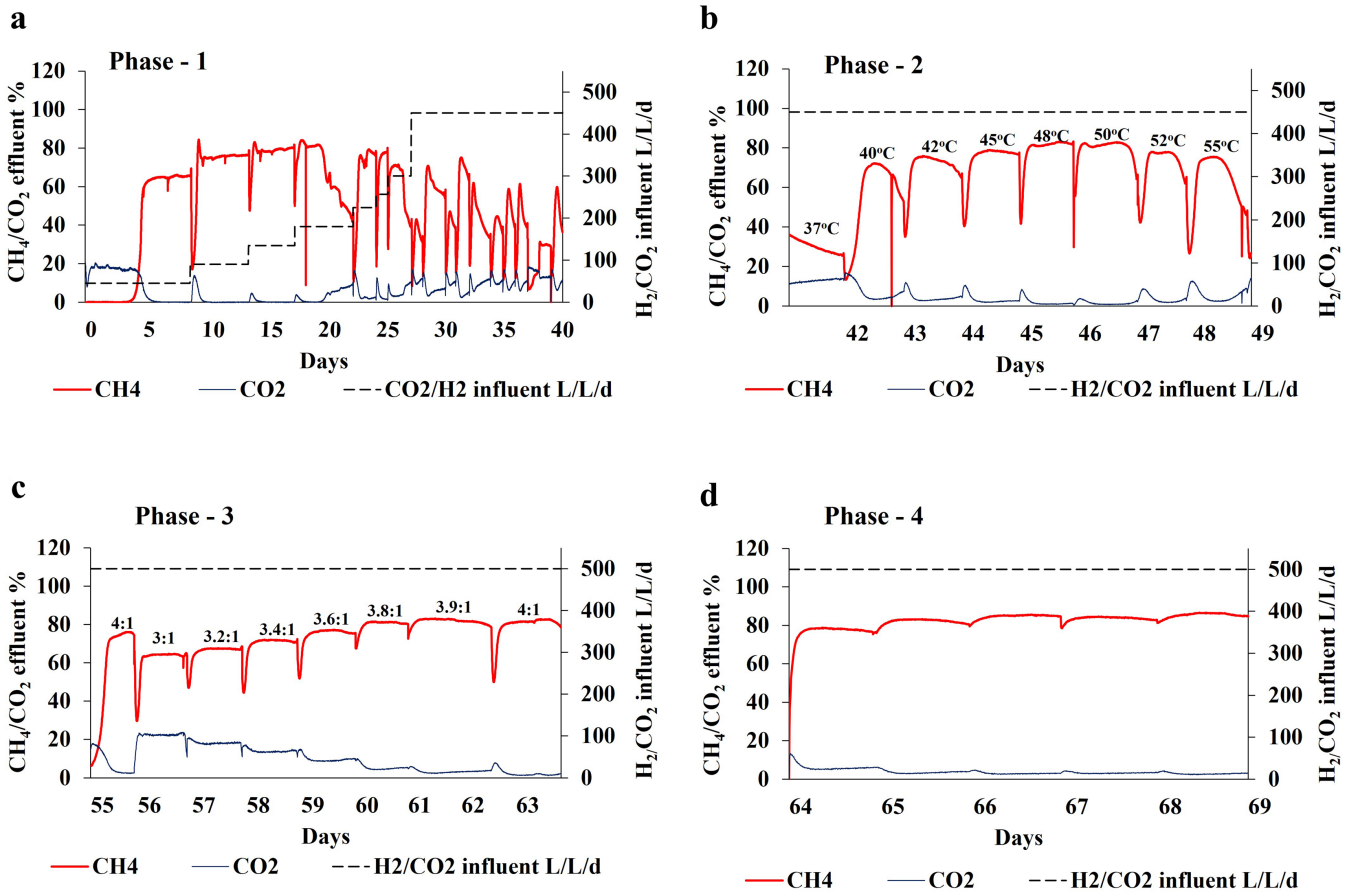
Methane yield from the *ex-situ* biomethanation process using autoclaved anaerobic sludge as inoculum, in Phase 1–4: **(a)** Phase 1, methane yield under varying H_2_: CO_2_ (4:1) throughputs at 37 °C; **(b)** Phase 2, methane production during biomass temperature conditioning. The temperature was gradually increased, as illustrated in the chart. The H_2_: CO_2_ (4:1) flow rate was maintained at 450 L/L/d; **(c)** Phase 3, methane yield under varying H_2_ fractions in the H_2_: CO_2_ gas mixture (total flow rate 500 L/L/d), with specific gas ratios indicated in the chart. The temperature was maintained at 50 °C; **(d)** Phase 4, methane production by the conditioned and adapted biomass under a constant H_2_: CO_2_ (4:1) throughput of 500 L/L/d at 50 °C. The relative error associated with gas measurements was ±2%.

Figure 3a illustrates the H₂/CO₂ inflow rate along with the CO₂ and CH₄ concentrations in the output gas for Phase 1 of the experiment. The CH₄ production began between days 4 and 5, peaking at 65%. This CH_4_ level remained stable until day 9, with the H₂/CO₂ input held constant at 45 L/L/d. Following a first washout procedure and an increase in throughput to 90 L/L/d, the CH₄ concentration rose to 76%, between days 10 and 13. A further increase in gas input to 135 L/L/d, under similar conditions led to an additional rise in CH₄ concentration, reaching 80% and sustaining until day 18. Nonetheless, after day 18, following additional washouts and increased H_2_/CO_2_ inputs, CH_4_ values in the effluent gas dropped significantly. The system became highly unstable, with CH_4_ content falling below 60%, particularly between days 28 and 40, when the H_2_/CO_2_ throughput was sustained at 450 L/L/d.

**Figure 4:**
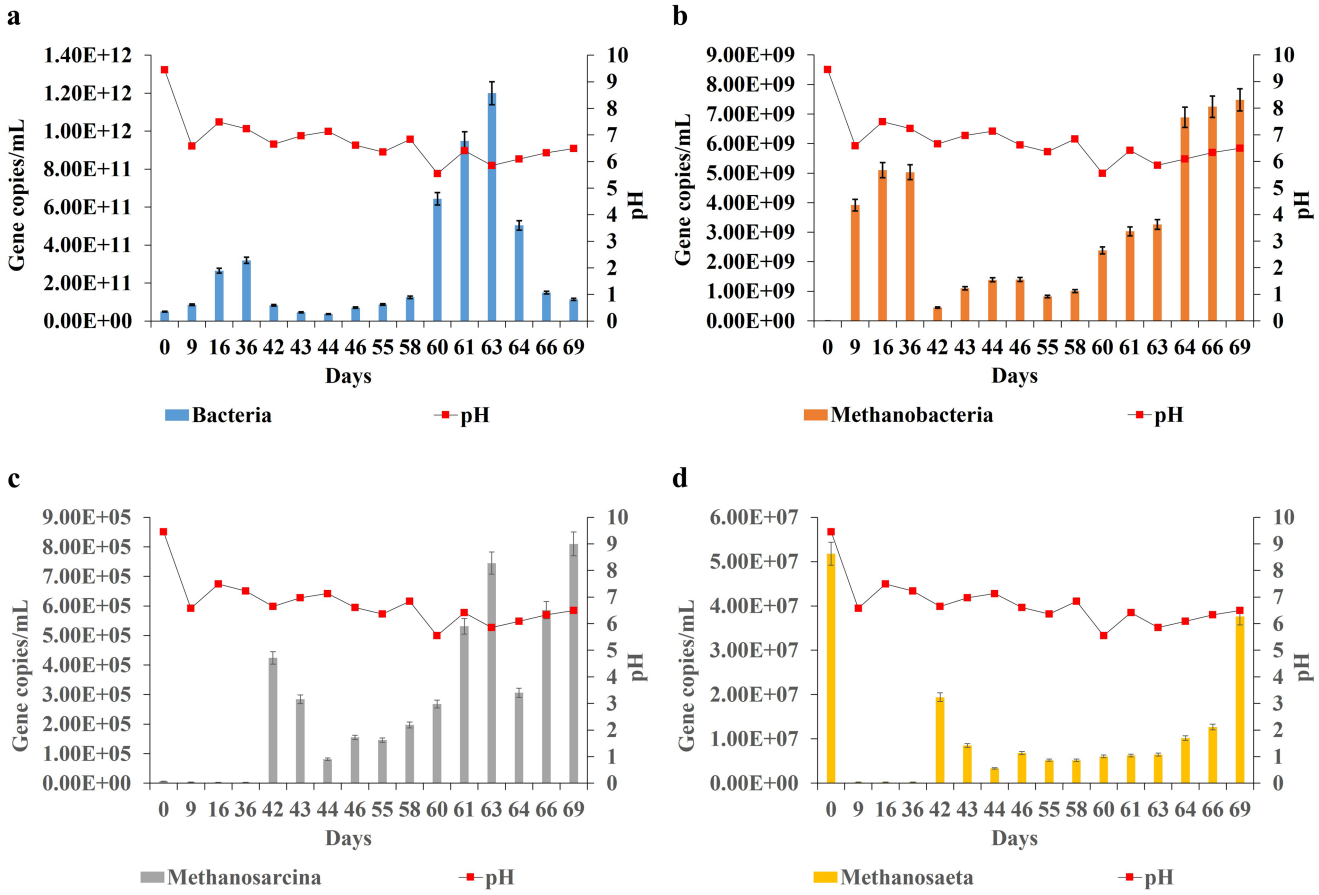
qPCR results displaying gene copies/mL of **(a)** bacteria (blue columns), **(b)** *Methanobacteria* (orange columns), **(c)** *Methanosarcina* (grey columns), and **(d)** *Methanosaeta* (yellow columns) from samples across four phases (days 0–69). The primary vertical axis shows gene copies/mL, while secondary vertical axis indicates pH of samples.

The sharp decline in CH_4_ levels and process instability, particularly at H_2_/CO_2_ throughput of 450 L/L/d, was likely due to the decreased activity of *Methanosaeta* and *Methanosarcina* (Figure 4; Days 0–36). By day 36, the populations of *Methanosaeta* and *Methanosarcina* had decreased by approximately 211 and 2.3 times, respectively, compared to their initial levels on day 0 (Figure 4; Days 0–36). On the contrary, bacteria and *Methanobacteria* increased by approximately 6.4 and 25,404-fold, respectively (Figure 4; Days 0–36). Despite the remarkable rise in the gene copies of hydrogenotrophic *Methanobacteria*, their metabolic activity was likely suppressed by increased competition for H_2_ and CO_2_ from acid-producing bacteria. Simultaneously, a sharp reduction in *Methanosaeta*, an important acetate consuming archaeon, further contributed to the decline in CH_4_ production.

**Figure 5:**
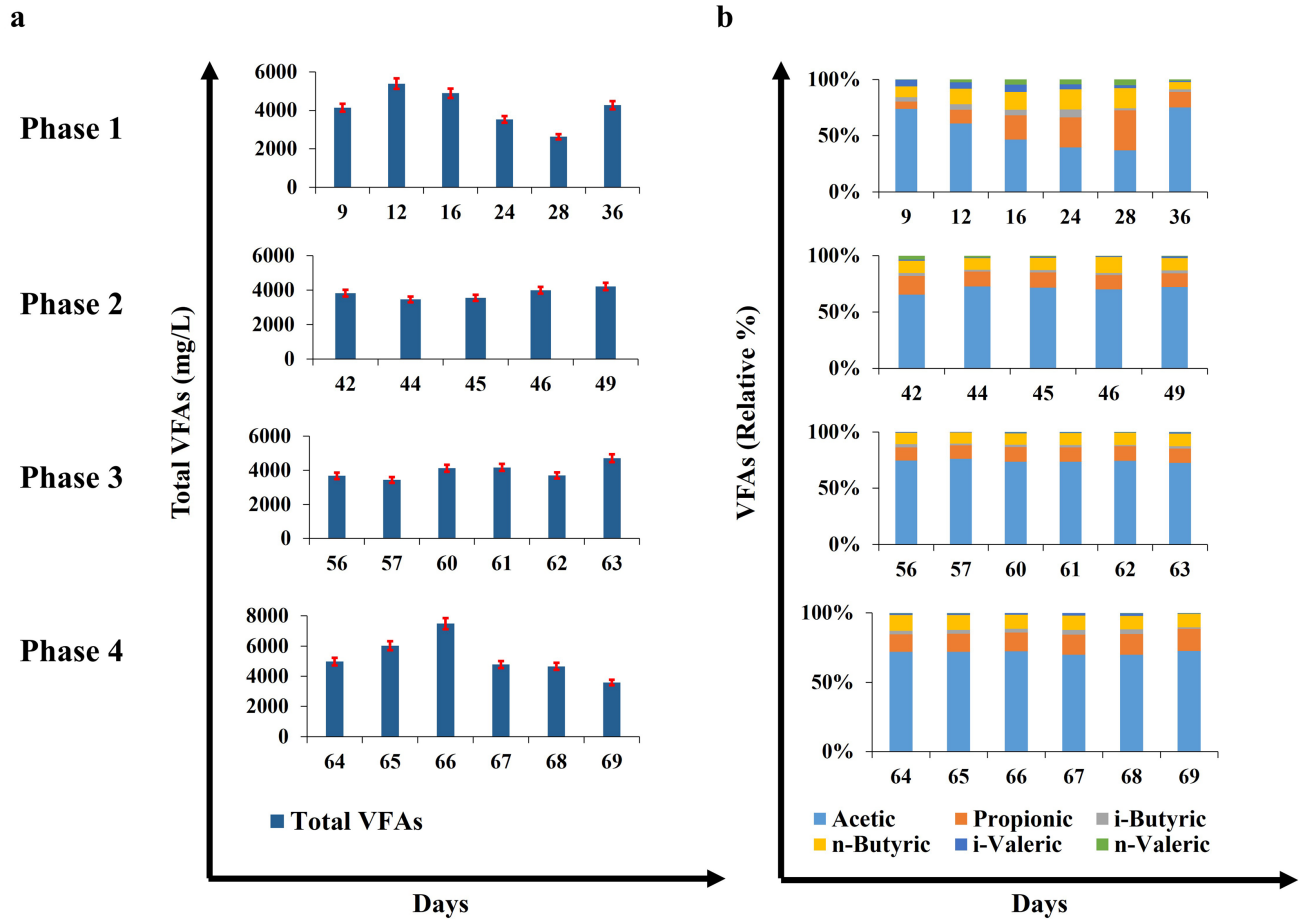
**(a)** Total volatile fatty acids across four phases. **(b)** Relative percentage of acetic, propionic, *n*-butyric, *i*-butyric, *n*-valeric, and *i*-valeric in the total volatile fatty acid. The standard error of the mean for the relative composition was generally within ±5% of the mean across all measurements (*n* = 3).

As shown in Figure 5a (phase 1), the total VFAs measured on days 9, 12, 16, 24, 28, and 36 were 4.1, 5.4, 4.9, 3.5, 2.6, and 4.2 g/L, respectively; VFA concentrations were measured immediately before washout, capturing peak accumulation prior to dilution or biomass replacement. This accumulation of VFAs likely resulted from a kinetic imbalance between acid-producing bacteria and acid consumer *Methanosaeta* (Logroño et al., 2020). Specifically, on days 16, 24, and 28, propionic acid made up 22, 27, and 35% of the total VFAs, while acetic acid accounted for 46, 40, and 40%, respectively. Also, butyric acid comprised 16, 18, and 18% of the total VFAs, respectively (Figure 5b; phase 1).

The accumulation of VFAs such as acetate, propionate, and butyrate can inhibit the biomethanation process (Capson-Tojo et al., 2016). Acetate plays a key role as its degradation by methanogens facilitates the breakdown of other VFAs through syntrophic oxidation (Lv et al., 2020). Propionate is more difficult to degrade and can become toxic to microbes at increased levels (Wang et al., 2020). Excessive amounts of acetate and propionate can further impair methanogenesis by promoting resistant bacteria that can outcompete methanogens for shared substrates (Regueiro et al., 2015). Additionally, propionate, being in a highly reduced state, must first be converted into CH_4_ precursors such as acetate, formate, or hydrogen by acetogens (Gallert and Winter, 2005). This process relies on tightly coupled syntrophic interactions that require low H_2_ partial pressure and low acetate concentrations (McCarty and Smith, 1986). However, at elevated H_2_/CO_2_ throughput of 450 L/L/d (days 28–40), a marked reduction in *Methanosaeta* gene copies, key acetoclastic methanogens responsible for maintaining low acetate levels, likely disrupted these syntrophic relationships. Elevated H₂ partial pressure likely imposed thermodynamic constraints on propionate-oxidising bacteria, impairing propionate degradation. Despite 50% washouts during this period, CH₄ production did not improve, indicating disruption of syntrophic balance.

Prior experiments conducted using unautoclaved sludge under comparable conditions resulted in rapid acidification and process failure at H_2_/CO_2_ throughputs exceeding 100 L/L/d. These observations are referenced here for contextual comparison and are being reported elsewhere; relevant supporting data is included as Supplementary material 1.

At this stage, it remained unclear whether the instability was primarily caused by biomass shock due to the high H₂/CO₂ throughput (450 L/L/d) or by the system’s limited capacity to process increased gas inputs at 37 °C. This suggests that thermophilic operation may be required to enhance performance under the high throughput ex-situ biomethanation conditions.

#### 3.2.2 Phase 2: Temperature conditioning of biomass for improved CH_4_ production

During Phase-2 (days 41–49) the temperature was gradually increased by 2–3 °C every 24 h, starting at 37 °C and reaching 55 °C. These temperature adjustments were made after each 50% culture washout (Figure 3b).

Figure 3b shows that, unlike the mesophilic systems described in Phase 1 (Figure 3a) which became unstable due to increasing H₂/CO₂ throughput, the thermophilic system-maintained stability. Operating at temperatures between 40 and 55 °C, it effectively handled H₂/CO₂ loading rates of 450 L/L/d, resulting in a significant improvement in both CH₄ production and overall process stability (Figure 3b). The highest levels of stability and CH₄ content of 82% in the output gas was observed at 48 and 50 °C (Figure 3b). Also, as the stirring speed and gas throughput in both phases 1 and 2 was sustained at 2500 rpm and 450 L/L/d, respectively, the enhanced biomethanation observed in phase-2 is likely due to the elevated temperature. This observation is consistent with Zhu et al. (2019), which demonstrated that thermophilic conditions enhance H_2_ utilisation by hydrogenotrophic methanogens, particularly at high stirring rates.

**Figure 6:**
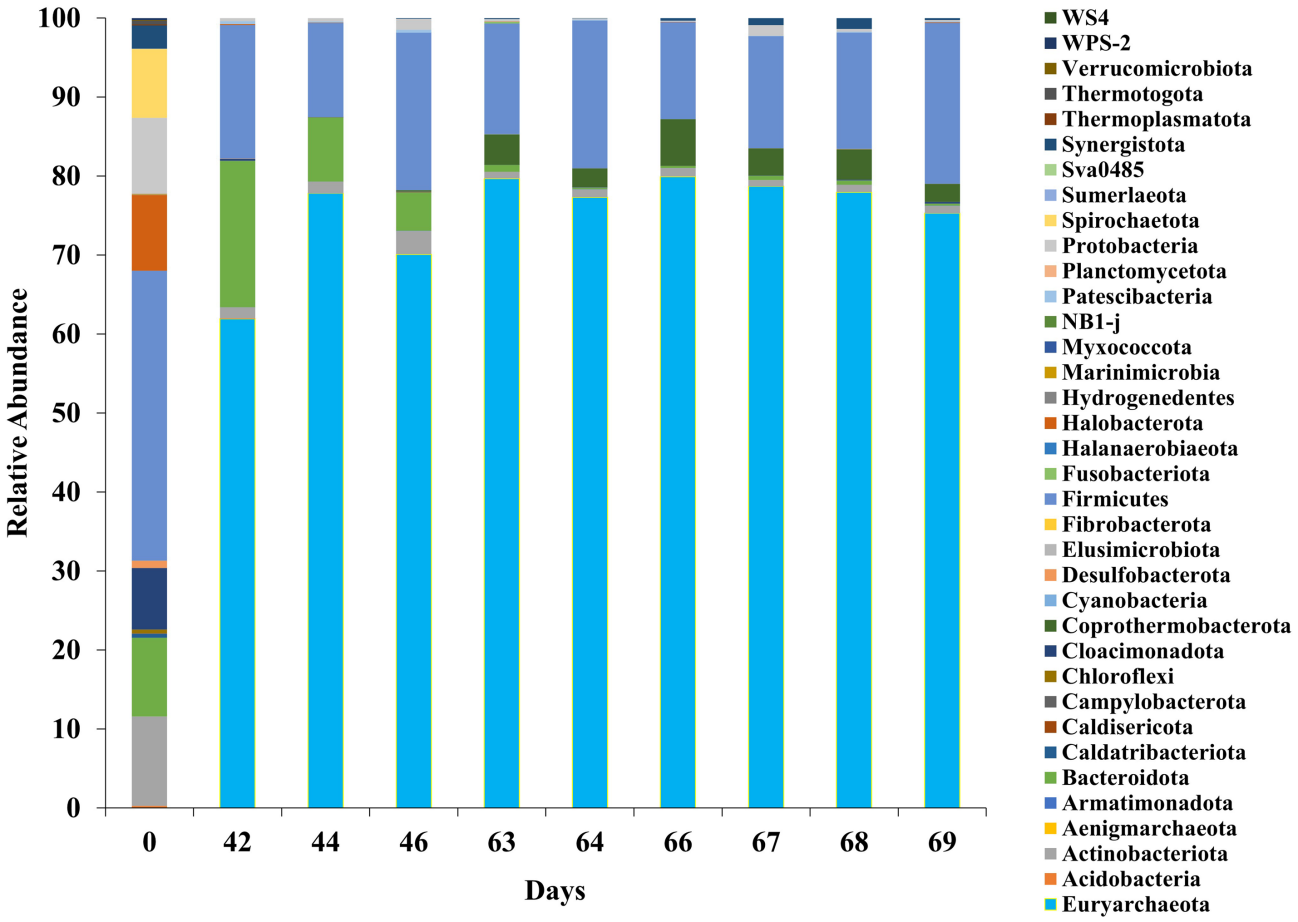
16S rRNA analysis showing the relative abundance of microbial phyla in samples collected on days indicated on the primary horizontal axis.

During days 45 to 47, as temperatures reached 48–50 °C, 16S rRNA sequencing identified a 0.2% abundance of *Coprothermobacter* sp., a thermophilic, hydrogen-producing bacterium (Figure 6; day 46). These microorganisms, known to flourish at temperatures between 50 and 70 °C and form symbiotic associations with hydrogenotrophic methanogens, likely contributed to the noted increase in CH_4_ production and process stability at 50 °C (Gagliano et al., 2015). Nonetheless, the microbial community was predominantly composed of *Euryarchaeota* and *Firmicutes*, which made up roughly 70 and 20% of the total microbial abundance, respectively (Figure 6; day 46). Additional phyla observed included *Bacteroidota*, *Actinobacteriota*, *Proteobacteria*, *Patesibacteria*, and *Synergistota*.

The abundance in *Firmicutes* resulted in synthesis of VFAs via homoacetogenic pathway under H_2_/CO_2_ loading of 450 L/L/d, the total mean values varied between 3.4 and 4.2 g/L (Figure 5a; phase 2). Acetic acid dominated the VFA profile, comprising an average 70% of the total concentration, while propionic and n-butyric acids contributed approximately 13.5 and 11.5%, respectively (Figure 5b; phase 2). Compared to mesophilic conditions (Section 3.2.1) the higher temperature resulted in reduced levels of propionic and n-butyric acids. This shift is likely attributable to the resurgence of *Methanosaeta* activity (Figure 4d; Days 42–46) and potential enhanced syntrophic oxidation of propionic and n-butyric acids to acetic acid, a process that is favoured under thermophilic conditions (Westerholm et al., 2022; Nikitina et al., 2023).

The gene copy numbers of microbial populations were assessed using qPCR, as shown in Figure 4. On day 42, shortly after incubation at 37 °C, the gene copy numbers were as follows: 8.3 × 10^10^ for bacteria, 4.5 × 10^8^ for *Methanobacteria*, 4.2 × 10^5^ for *Methanosarcina*, and 1.9 × 10^7^ for *Methanosaeta* (Figure 4). When the temperature was raised to 42 °C (between days 43 and 44), a decline was observed in most populations, with gene copy numbers decreasing to 3.7 × 10^10^ for bacteria, 8.2 × 10^4^ for *Methanosarcina*, and 3.3 × 10^6^ for *Methanosaeta*, while *Methanobacteria* showed an increase to 1.4 × 10^9^ (Figure 4; Day 44). This indicates that the biomass experienced temperature shock, with *Methanobacteria* being the exception as it adapted and increased its activity. However, at 48 °C (between days 45 and 46), recovery was observed in bacterial and *Methanosaeta* populations with gene copy numbers reaching 7 × 10^10^ and 3.8 × 10^6^, respectively. *Methanobacteria* maintained a stable population at 1.4 × 10^9^ while *Methanosarcina* exhibited minimal activity with a gene copy number of 1.5 × 10^5^ (Figure 4; Day 46). These results indicate that a sudden increase in temperature disrupts microbial populations, with different species exhibiting varying abilities to recover and adapt.

Nonetheless, this study showed that biomass could effectively adapt to a high H_2_: CO_2_ (4:1) throughput of 450 L/L/d by gradually increasing the temperature to 50 °C. This temperature conditioning enhanced microbial tolerance and stability under elevated gas throughput. While higher temperatures of 52 and 55 °C reduced CH₄ content to 76 and 73%, respectively, 50°C achieved the optimal balance between process stability and CH₄ production, yielding 82% (Figure 3b). This performance surpassed that of mesophilic conditions at 37 °C under similar throughputs (see Section 3.2.1 and Figure 3a).

#### 3.2.3 Phase 3: Optimising the H₂/CO₂ ratio to stabilise CH₄ production at 500 L/L/d throughput

The third phase sought to further increase the H_2_/CO_2_ throughput to 500 L/L/d while sustaining a 50 °C temperature and a stirring rate of 2,500 rpm (Figure 3c). When the loading rate was increased to 500 L/L/d on day 55, the CH_4_ concentration in the output gas dropped to 74% (Figure 3c). This decrease suggested a reduction in system stability, possibly due to the biomass needing time to adapt to the higher H_2_/CO_2_ levels. To address this issue, a 50% culture washout was performed on day 56 and the H_2_: CO_2_ ratio was temporarily lowered to 3:1 (Figure 3c). Although this adjustment stabilised the system, it resulted in a further decrease in CH_4_ content to 64%, likely due to reduced H_2_ availability. Subsequently, H_2_ content was progressively increased, as illustrated in Figure 3c. By day 63, the H_2_: CO_2_ ratio was restored to 4:1, and the system exhibited improved stability, producing an output gas with 82% CH_4_. This outcome demonstrated the successful adaptation of the biomass to the higher H_2_/CO_2_ loading rate of 500 L/L/d at 50 °C.

The microbial community abundance on day 63 was characterised at the phylum level (Figure 6), showing 80% *Euryarchaeota* and 14% *Firmicutes*. Notably, *Coprothermobacterota* increased to 4%, up from just 0.2% in the phase-2 (section 3.2.2). Other phyla, including *Bacteroidota*, *Actinobacteriota*, *Proteobacteria*, *Fusobacteriota*, and *Synergistota*, comprised 0.87, 0.84, 0.3, 0.2, and 0.1% of the total microbial abundance, respectively (Figure 6; day 63). The thermophilic family *Coprothermobacteraceae*, previously classified under *Firmicutes*, has been reassigned to the newly established phylum *Coprothermobacterota* (Steiniger et al., 2023). Members of this phylum, particularly *Coprothermobacter*, are key syntrophic acetate-oxidising bacteria (SAOB).

To evaluate the microbial response to increased H₂/CO₂ throughput, gene copy numbers of key microbial groups were quantified by qPCR between days 55 and 63. On day 55, increasing the H₂: CO₂ (4:1) loading rate to 500 L/L/d, compared to day 46, led to a rise in bacterial gene copy numbers (8.71 × 10^10^), while those of *Methanobacteria* (8.26 × 10^8^), *Methanosarcina* (1.46 × 10^5^), and *Methanosaeta* (5.2 × 10^6^) declined (Figure 4). This shift also led to a reduction in CH₄ yield (74%) and resulted in slight system instability (Figure 3c; day 55). However, a gradual adjustment of H₂ levels over 24-h intervals, while maintaining the 500 L/L/d of H_2_/CO_2_ throughput, facilitated the recovery of methanogens, as indicated by increased gene copy numbers (Figure 4; Days 55–63).

By day 63, gene copy numbers had increased significantly compared to day 55, showing a ~ 14-fold rise for bacteria, 4-fold for *Methanobacteria*, 5-fold for *Methanosarcina*, and 1.2-fold for *Methanosaeta* – reaching 1.2 × 10^12^, 3.26 × 10^9^, 7.45 × 10^5^, and 6.46 × 10^6^, respectively (Figure 4). Hence, by gradually adjusting the H₂ throughput, the system stability improved, and the CH₄ content in the effluent gas increased from 74% on day 55 to 82% on day 63 (Figure 3c). Additionally, pH measurements during this period fluctuated between 6.8 and 5.6, reaching 5.9 on day 63 (Figure 4; Days 55–63), indicating microbial adaptation to acidic conditions.

Throughout this phase, the average concentration of VFAs was approximately 4 g/L (Figure 5a; phase 3). Acetic acid dominated the composition at 74%, followed by propionic acid (12%) and butyric acid (11%) (Figure 5b; phase 3). The remaining VFAs, present in trace amounts, had a minimal impact on the overall composition (Figure 5b; phase 3). The production of VFAs can be attributed to the abundance of *Firmicutes*, which are involved in various metabolic processes that break down carbohydrates and fatty acids, including homoacetogenesis via the Wood-Ljungdahl pathway and syntrophic acetate oxidation. This could account for the high presence of this phylum in environments with elevated H₂ levels. Additionally, the substantial amounts of acetate facilitated the proliferation of *Methanosaeta* (Figure 4; Days 55–63), an obligate acetoclastic methanogen with a strong affinity for VFAs.

Furthermore, increased H_2_ supply rates led to an enrichment of hydrogenotrophic *Methanobacteria* (Figure 4; Days 55–63) that produce CH_4_ using H_2_ as electron donor to reduce CO_2_. The rise of *Methanosarcina* (Figure 4; Days 55–63) could be attributed to both high VFA and H_2_ loading rates, as this organism is capable of both hydrogenotrophic and acetoclastic methanogenesis (Wahid et al., 2019). Also, the rise in the relative abundance of *Coprothermobacter* likely supported its hydrogenotrophic partners (Mahdy et al., 2019), potentially contributing to the enhanced stability of the process performance, as shown in Figure 3c.

#### 3.2.4 Phase 4: Performance of conditioned biomass at 500 L/L/d of H_2_: CO_2_ throughput

Phase 4 (days 64–69) evaluated the robustness and productivity of the adapted biomass under sustained H_2_: CO_2_ (4:1) throughput (500 L/L/d) at 50 °C. This phase also investigated whether the washout volume could be reduced to below 50% without affecting the recovery speed of the biomethanation process. By minimising the washout volume, the study aimed to retain sufficient microbial biomass to support rapid recovery and optimal process performance. Moreover, as hydrogenotrophic methanogenesis also produces two moles of water for each mole of CH_4_ generated, the washout procedure accounted for this dilution effect while restoring the system to its original working volume.

On day 64, a 25% washout using autoclaved digestate was conducted, leading to 78% CH₄ in the output gas (Figure 3d). Subsequent washouts were performed every 24 h. Notably, CH₄ levels in the output gas steadily increased with each washout, reaching 86% by day 69. This trend coincided with improved system stability, as illustrated in Figure 3d. The volumetric gas effluent was also quantified, the total gas output was 126 L/L/d, comprising 108 L/L/d of CH_4_ and 4 L/L/d of CO_2_, with the remaining 14 L/L/d primarily composed of H_2_ (see Supplementary material 2). While the experiment concluded on day 69, continued 25% washouts every 24 h could potentially had pushed CH₄ content beyond 90%. Additionally, as in this phase, the washout volume was reduced from 50 to 25% compared to earlier phases, further reductions may be achievable as the biomass develops greater tolerance to conditions at 500 L/L/d throughput and 50 °C. Also, as biomass adaptation improves, decreasing the frequency of washouts could enhance scalability. However, this aspect is yet to be tested.

Overall, the 25% washouts had minimal impact on CH₄ production as methanogenic activities recovered quickly, demonstrating the resilience and adaptability of conditioned biomass to significantly higher H_2_: CO_2_ throughputs, at 50 °C (Figure 3d).

The microbial community structure was analysed at the phylum level between days 64 and 69. The results indicate that *Euryarchaeota*, *Firmicutes*, and *Coprothermobacterota* dominated, accounting for an average relative abundance of 78, 16, and 4%, respectively (Figure 6). Moreover, qPCR analysis on day 64 showed gene copy numbers of 5 × 10^11^ for bacteria, 6.9 × 10^9^ for *Methanobacteria*, 3 × 10^5^ for *Methanosarcina*, and 1 × 10^7^ for *Methanosaeta* (Figure 4). By day 69, bacterial gene copy number had decreased to 1.2 × 10^11^, while *Methanobacteria*, *Methanosarcina*, and *Methanosaeta* increased to 7.5 × 10^9^, 8 × 10^5^, and 3.8 × 10^7^, respectively (Figure 4). The decreased bacterial activity was also evident in the production of VFAs, which was measured at approximately 5 g/L on day 64, dropping to 3.5 g/L by day 69 (Figure 5a; phase 4). At this point, the VFAs comprised 73% acetate, 16% propionic acid, and 10% n-butyric acid, with other VFAs present in negligible amounts (Figure 5b; phase 4). The rise in CH_4_ levels, from 78% on day 64 to 86% on day 69, can be directly linked to the increased activity of both hydrogenotrophic and acetoclastic methanogens, as demonstrated in Figure 4 (Days 64–69).

The results confirm that higher temperatures and elevated H_2_/CO_2_ loading rates lead to a reduction in microbial diversity. Microbes unable to adapt to these conditions seem either to enter dormancy or die. The dominance of *Euryarchaeota* (78%), *Firmicutes* (16%), and *Coprothermobacterota* (4%) (Figure 6; Day 69), together comprising 98% of the microbial community, indicates that a temperature of 50 °C and a H_2_: CO_2_ input of 500 L/L/d promoted a highly specialised microbial ecosystem.

Overall, in the final phase, the system exhibited a balanced microbial community, with *Firmicutes* producing VFAs, *Coprothermobacterota* converting acetate to H₂ and CO₂, and *Methanosaeta* converting acetate to CH₄ and CO₂. The high H₂: CO₂ throughput at 50 °C favoured hydrogenotrophic *Methanobacteria*, while *Methanosarcina*, though less abundant, contributed to system stability through both hydrogenotrophic and acetoclastic methanogenesis.

## 4 Conclusion

This study demonstrates robust high-throughput ex-situ thermophilic biomethanation using autoclaved sludge, addressing key limitations associated with mixed microbial systems. Notably, functional methanogenic communities were shown to withstand autoclave treatment (121 °C, 1 h) and be selectively enriched under thermophilic conditions. While mesophilic operation (37 °C) at high H₂/CO₂ loading (450 L/L/d) resulted in instability and low CH₄ content (<60%), thermophilic conditioning at 50 °C significantly enhanced performance, achieving 82% CH_4_. Further increasing throughput to 500 L/L/d at 50 °C yielded 86% CH_4,_ with potential to exceed 90%, representing one of the highest performances reported for CSTR based biomethanation at atmospheric pressure.

Microbial analysis confirmed the development of a specialised consortium dominated by *Euryarchaeota*, with supporting thermophilic bacterial partners, indicating effective selection of hydrogenotrophic methanogenesis and suppression of competing pathways. These findings highlight the critical role of temperature-driven microbial selection and process control in enabling stable operation under high gas loading.

Overall, sustained high conversion efficiencies at elevated gas throughputs reduce reactor footprint and improve process economics, supporting ex-situ biomethanation for renewable energy storage and carbon utilisation in decentralised Bio-P2G systems. However, as the system was operated with autoclaved digestate, long-term stability under non-sterile conditions typical of industrial operation remains to be validated, and future work should assess robustness under continuous, non-sterile feeding. Within this context, thermophilic operation at 50°C is most viable when integrated with existing low-grade heat streams and should be considered as part of a coupled energy system, rather than an isolated process requirement.

## Data Availability

The original contributions presented in the study are included in the article/Supplementary material, further inquiries can be directed to the corresponding author/s.
