# Supplementary figures and images for: Development of a mixed microbial culture for robust high-throughput ex-situ thermophilic biomethanation
Source: Front Microbiol. 2026 May 28;17:1840981. doi: 10.3389/fmicb.2026.1840981 (PMC13256089; doi:10.3389/fmicb.2026.1840981)

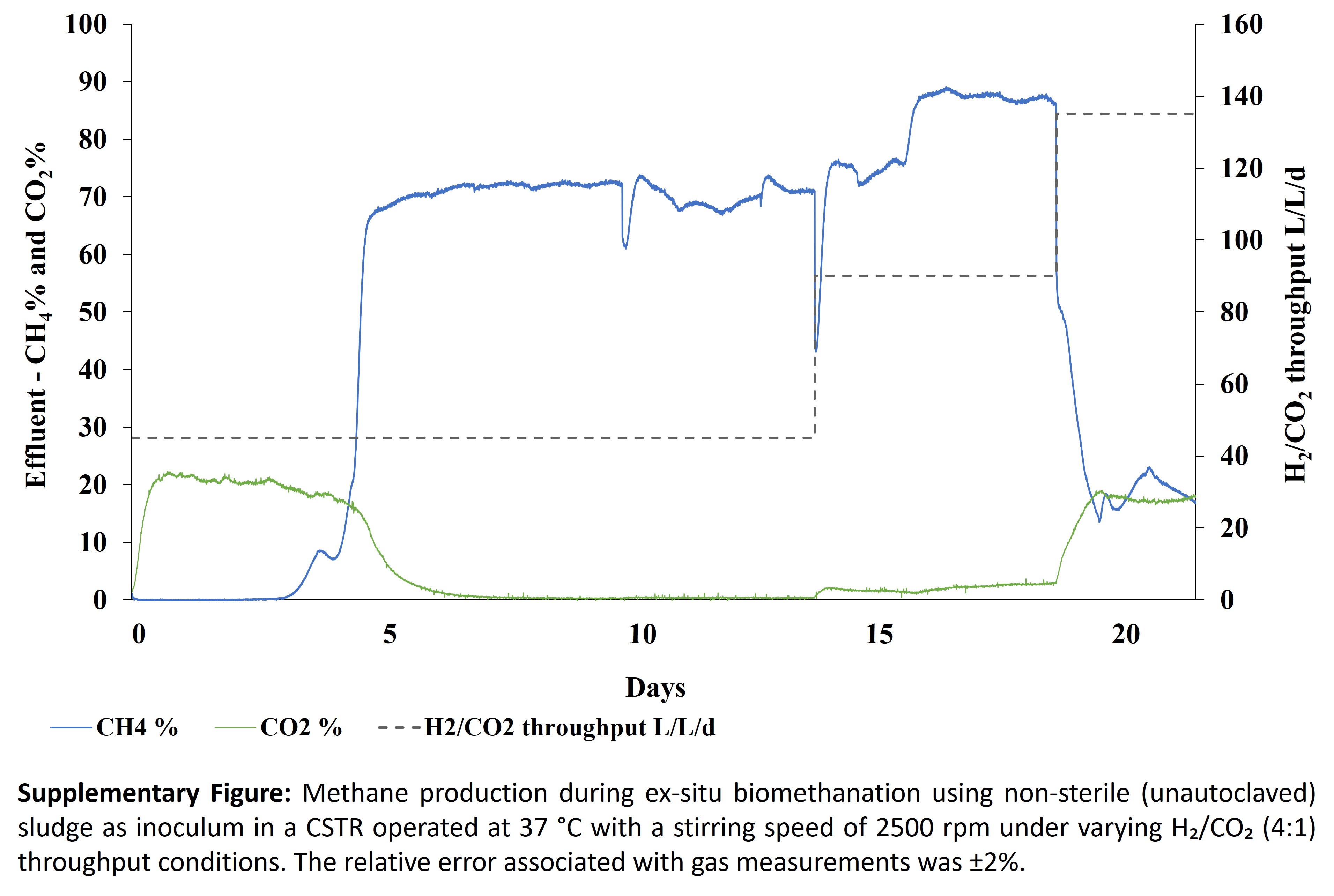

Supplement: Supplementary file 1 [file Image_1.jpg]

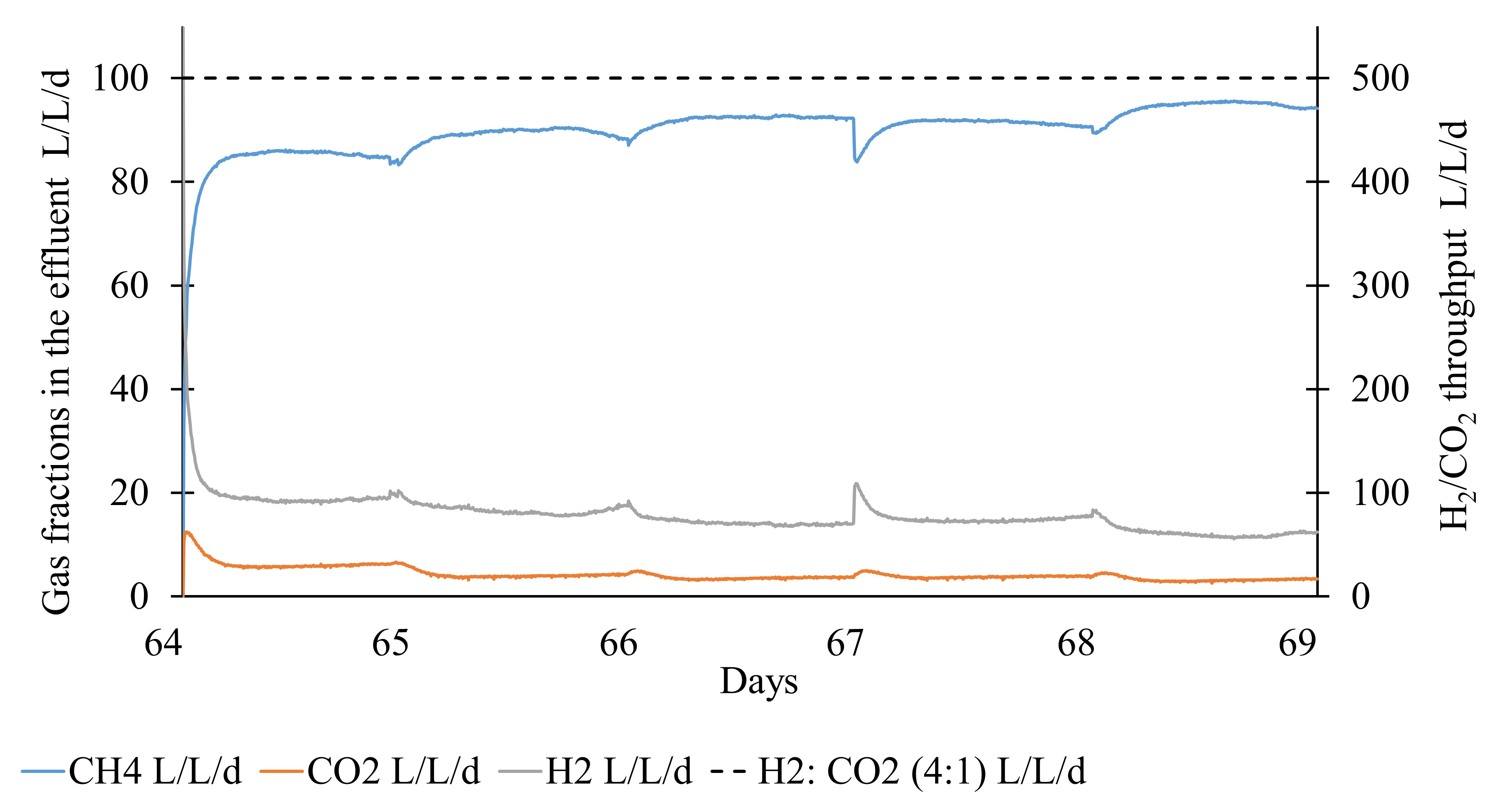

Supplement: Supplementary file 2 [file Image_2.jpg]
